# Potential Cytoprotective and Anti-Apoptotic Effect of Metamizole Alone and in Combination with Cytostatic Drugs Observed In Vitro in Canine (D-17) and Human (U-2 OS) Osteosarcoma Cell Lines

**DOI:** 10.3390/biomedicines12030571

**Published:** 2024-03-03

**Authors:** Dominik Poradowski, Aleksander Chrószcz, Radosław Spychaj, Vedat Onar

**Affiliations:** 1 Department of Biostructure and Animal Physiology, Division of Animal Anatomy, Faculty of Veterinary Medicine, Wroclaw University of Environmental and Life Sciences, ul. Kożuchowska 1, 51-631 Wrocław, Poland; 2 Department of Fermentation and Cereals Technology, Faculty of Biotechnology and Food Science, Wroclaw University of Environmental and Life Sciences, ul. J. Chełmońskiego 37, 51-630 Wrocław, Poland; radoslaw.spychaj@upwr.edu.pl; 3 Osteoarchaeology Practice and Research Centre & Department of Anatomy, Faculty of Veterinary Medicine, Istanbul University-Cerrahpaşa, 34320 Avcılar, Istanbul, Türkiye

**Keywords:** osteosarcoma, cell line, metamizole, human, canine, cytoprotective effect

## Abstract

Metamizole (dipyrone) is still a frequently used drug in human and veterinary medicine, especially for pain relief, sometimes also in osteosarcoma treatment. It has a very strong analgesic and antipyretic effect and weaker anti-inflammatory activity. Additionally, it has spasmolytic properties. It is used in many species, including dogs, cats, pigs, cattle, horses, and humans, in Europe, Canada, and South America. The aim of this study was to assess a potential synergism of metamizole as a non-steroidal anti-inflammatory drug with routinely used cytostatics (cisplatin, carboplatin, doxorubicin, and etoposide). In vitro studies were performed on a canine osteosarcoma D-17 cell line and a human U-2 OS cell line. We used the MTT method to assess cell viability, TUNEL staining to assess proapoptotic effects, and propidium iodide to analyse the cell cycle (potential arrest). The obtained results showed that metamizole at 50 μg/mL has potential cytoprotective and anti-apoptotic effects. Metamizole administered simultaneously with cytostatic drugs reduced their cytotoxic effect, which may suggest that such a combination of drugs used in the treatment of osteosarcoma may significantly reduce therapy effectiveness.

## 1. Introduction

Osteosarcoma (OSA) is a malignant bone tumour of mesenchymal origin occurring in animals, including humans, with a highly diverse histological structure. When describing this neoplasm, the classification proposed by the World Health Organisation (WHO) is routinely used, in which the main criteria include type of focus, type of matrix, location, and degree of malignancy. This classification also includes very rare types of OSA, such as small cell and telangiectatic, which resemble angiosarcoma in appearance [1,2]. Radiologically, osteosarcomas can be classified as solid, sclerotic, or mixed, and, due to their cellular composition, as single, composite, and pleomorphic [2]. Osteosarcoma occurring in animals is very similar to that occurring in humans, primarily in its morphological structure, location, and predisposing factors [3]. Both in humans and dogs, the aetiology and pathogenesis of OSA are not fully known. However, there are several genetic and molecular factors associated with its occurrence, including overexpression of COX-2 and metalloproteinases as well as disorders in the synthesis of ezrin protein. These compounds, together with radixin and moesin, belong to the ERM protein family and act as molecular connectors between proteins anchored in the cell membrane and actin filaments [4,5,6]. In humans, osteosarcoma is the most frequent bone neoplasm after multiple myeloma [7], but in dogs, it is the most frequently diagnosed bone neoplasm (approximately 80–95% of cases). In dogs, osteosarcoma most often occurs in the long bones of adult animals of large and giant breeds (Boxer, German shepherd, St. Bernard, etc.) [8,9], but it may also occur in puppies [8,10,11,12,13]. It quickly spreads to the bordering soft tissues and often metastasises to the regional lymph nodes, lungs, other bones, skin, or liver [4,14,15].

In cases of OSA located in the long bones, the most common symptoms include pain and swelling of the affected area, lameness of varying degree, and pathological fractures. Neoplastic cachexia syndrome is very common in patients with advanced disease. Osteosarcoma is not accompanied by any paraneoplastic syndromes. In humans, as well as in dogs, this disease occurs more often in males than in females [7]. This fact can be explained by the body mass of an individual. This pathology is most often diagnosed in childhood and adolescence, with peak incidence at the age of 16. Another increase in the incidence of this type of neoplasm is observed in the elderly [16,17]. Clinical symptoms in humans also largely resemble those observed in dogs. OSA treatment in humans and dogs follows a similar schedule. After surgery, it is advisable to perform adjuvant chemotherapy. The cytostatic drugs most frequently used in adjuvant chemotherapy include cisplatin, carboplatin, doxorubicin, and etoposide. Regardless of the advancement and location of osteosarcoma and the type of implemented therapeutic protocol, it is important to use drugs that relieve the pain associated with the neoplasm. The WHO, in its manual issued in 1986 (which can also be used in dogs), recommends the use of non-steroidal anti-inflammatory drugs alone or in combination with opioids with a weak or moderate analgesic effect for mild to moderate pain. In the cases of severe, chronic pain, the use of opioids with a strong analgesic effect is recommended [18]. Radiotherapy is most often used in palliative treatment of patients with inoperable tumours or whose tumours have not been completely removed [19].

Metamizole was developed by German pharmaceutical company Hoechst AG in 1920, and in 1922, it entered mass production. The use of this drug, especially in humans, is controversial in many countries due to the risk of life-threatening side effects, e.g., gastrointestinal disorders, agranulocytosis, porphyria, anaphylactic reaction, or aplastic anaemia. For this reason, it has been removed from the market in many countries all over the world, e.g., in the United States, Australia, Sweden, Denmark, France, and Malaysia [20]. It is still widely used in Poland because it is effective, cheap, and available over the counter (OTC). In some European countries (Germany, Czech Republic, Slovakia, Finland, some Balkan countries etc.), metamizole is only a prescription drug. It has very strong analgesic and antipyretic effect, weaker anti-inflammatory properties, and also acts as a spasmolytic agent. It is used in many species, including humans, dogs, cats, pigs, cattle, and horses. Its mechanism of action involves the inhibition of COX-1, COX-2, and COX-3 activity and the stimulation of opioid receptors (indirect action) [21].

The aim of this work was to assess the potential anti-proliferative and proapoptotic effects of metamizole and its impact on the cell cycle, both alone and in combination with cytostatic drugs routinely used to treat OSA (cisplatin, carboplatin, doxorubicin, and etoposide) in in vitro-maintained canine (D-17) and human (U-2 OS) cell lines.

## 2. Materials and Methods

### 2.1. Cell Cultures

This study involved canine (D-17) and human (U-2 OS) osteosarcoma cell lines, both purchased from the American Type Culture Collection (Manassas, VA, USA). Cells of the selected lines were cultured in flasks with an area of 25 cm^2^ and 75 cm^2^ in an incubator with a constant 5% CO_2_ flow and a temperature of 37 °C (SANYO, model MCO-18AIC, Osaka, Japan). Standard, line-specific culture media were used: Eagle’s Minimum Essential Medium (ATCC, Manassas, VA, USA) for D-17 and McCoy’s 5A (ATCC, Manassas, VA, USA) for U-2 OS, supplemented with 10% foetal bovine serum (Sigma-Aldrich, Burlington, MA, USA), 4 nM L-glutamine (Sigma-Aldrich, Burlington, MA, USA), 100 U/mL penicillin, and 100 μg/mL streptomycin (Sigma-Aldrich, Taufkirchen, Germany).

### 2.2. Selected Drugs

Metamizole (met), doxorubicin (dx), carboplatin (carbo), and etoposide (et) were dissolved in the culture media. To prevent carboplatin from transitioning to its trans form, it was dissolved in 0.9% sodium chloride. The choice of drug concentrations used in this study was based on the maximum concentration they can achieve in serum; the type of solvent did not affect the range of tested concentrations. Table 1 presents the range of drug concentrations used in this study.

From all the tested concentrations, we selected those for which viability in the examined samples exceeded 50%, that is, was below the EC_50_ value (Table 2 and Table 3—see Section 3).

### 2.3. Assessment of Cell Viability with MTT Assay

Cells of the D-17 and U-2 OS lines were kept at a concentration of 3 × 10^3^/100 μL of the culture medium in 96-well culture plates (TPP, Trasadingen, Switzerland). After 24 h, the culture medium was removed and replaced with medium with selected concentrations of the investigated compounds and their combinations. MT staining was performed in accordance with the PN-EN ISO 10993-5 standard [23]. Four independent repetitions of the experiment were carried out and a mean of the obtained values was presented as a result. Mitomycin C solution served as a positive control, and clear culture medium served as a negative control. 

### 2.4. Assessment of Apoptosis with the TUNEL Method

To assess apoptosis, the ApopTag^®^ Peroxidase In Situ Apoptosis Detection Kit (Merck Millipore, Darmstadt, Germany) was used. The kit included an equilibration buffer, working-strength TdT enzyme, stop/wash buffer, anti-digoxigenin peroxidase conjugate, and DAB peroxidase substrate. D-17 and U-2 OS osteosarcoma cells at a density of 2 × 10^4^ cells suspended in 40 μL of a dedicated medium were placed on 10-well hydrophobic slides (Thermo Scientific, Waltham, MA, USA). After 24 h, the culture medium was replaced, and the cells were exposed to the investigated compounds alone and in combinations suspended in the culture medium (Table 1 and Table 2) for 72 h. Further procedures strictly followed the instructions included in the ApopTag® Peroxidase In Situ Apoptosis Detection Kit, up to the stage of staining cell nuclei, which was performed using a 1% haematoxylin solution (Merck Millipore, Darmstadt, Germany). At the final stage, the preparations were immersed for 30 s in 70% ethyl alcohol (Stanlab, Warsaw, Poland), then for 30 s in xylene (Stanlab, Warsaw, Poland), and then coverslips were attached using DPX (Thermo Scientific, USA).

The percentage of apoptotic cells was calculated in five randomly selected fields of vision at 40× magnification under an Olympus BX53 optical microscope (Olympus, Tokyo, Japan). The result was a mean of the results obtained from all examined fields. Independent evaluation of the immunohistochemical reaction was performed by two experienced researchers.

### 2.5. Cell Cycle Assessment with Propidium Iodide

Cells from the established canine and human osteosarcoma lines were adjusted to a density of 1 × 10^6^ per 2 mL of the culture medium and plated on sterile 6-well culture plates (TPP, Switzerland). After 24 h, the culture medium was substituted with fresh one, and the cells were exposed to the investigated compounds and their combinations at predefined levels (Table 1 and Table 2) for 72 h. After the incubation, the cells were exposed to a trypsin (0.25%) and EDTA (0.02%) solution to detach them from the bottom of the culture plate, then they were centrifuged and re-suspended in PBS. The number of living cells was assessed using trypan blue, and then the cells (1 × 10^6^) were transferred to a centrifuge tube containing 1 mL of PBS. The mixture was centrifuged for 5 min at 1200 rpm at 4 °C, and then the cells were suspended in 0.3 mL of PBS. To fix the cells and permeabilise their membranes, 0.7 mL of cold 70% ethyl alcohol was added dropwise while gently mixing the suspension to avoid the formation of cell conglomerates. The mixture was incubated on ice for one hour. Following the incubation, the cells were centrifuged, then washed once with PBS and centrifuged again. After pouring off the supernatant, the cells were suspended in 0.25 mL of PBS; 5 μL of RNAase A at a concentration of 10 mg/mL (Sigma-Aldrich, Burlington, MA, USA) was added, and the mixture was incubated for an hour at 37 °C. After this time, 10 μL of propidium iodide at a concentration of 1 mg/mL (Sigma-Aldrich, Burlington, MA, USA) was added. The samples, placed in cytometric tubes, were analysed using a flow cytometer (FACSCalibur, Becton Dickinson, Franklin Lakes, NJ, USA) equipped with an argon laser with an excitation wavelength of 488 nm. The results were processed using the WinMDI 2.9 package. The results are the mean of the results obtained from four independent repetitions of each of the preselected concentration of the tested drugs and combinations thereof.

### 2.6. Statistical Analysis

Statistical analysis was performed with StatisticaPL 13.0 software (StatSoft, Poland). For a detailed analysis, the Shapiro–Wilk test (data normality) and the Dunnett test (comparison of the obtained results with control) were used. In figures, horizontal lines within each box indicate the mean values. The lower and upper edge of each box indicate the mean value with standard deviation subtracted and added. Whiskers represent the maximum and minimum single data values. The significance level was set at *p* = 0.05.

## 3. Results

### 3.1. Cytotoxic Activity

Table 3 shows previously published EC_50_ values for the tested compounds [22].

**Figure 1:**
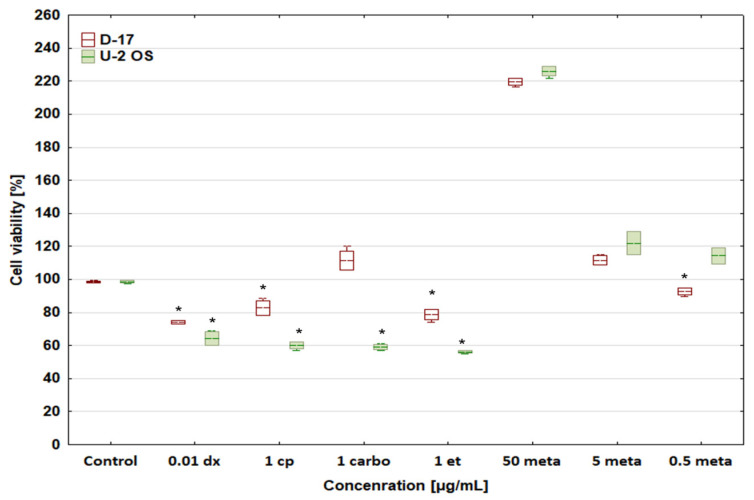
Effect of chosen cytostatic drugs and metamizole on cell viability of D-17 canine and human U-2 OS osteosarcoma cell lines. * values below control in the Dunnett test.

All tested cytostatic drugs at their selected concentrations reduced the viability of canine and human osteosarcoma cells (Figure 1). Human osteosarcoma cells were much more sensitive than canine ones. The only exception was carboplatin 1 μg/mL, under which the number of cells in the canine osteosarcoma D-17 sample increased as compared with the control, which may be due to the lower sensitivity of neoplastic canine cells vs human ones. The most interesting fact is that metamizole at 50 μg/mL strongly stimulated the viability of both neoplastic cell lines. 

**Figure 2:**
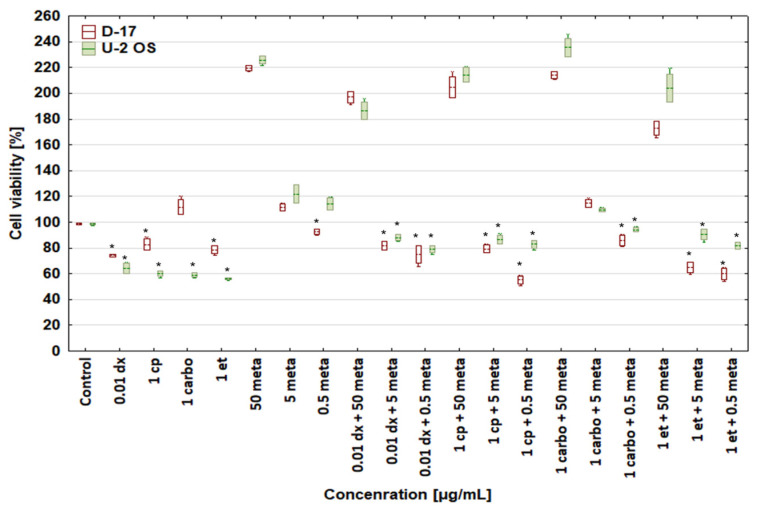
Effect of combinations of doxorubicin (dx), cisplatin (cp), carboplatin (carbo), and etoposide (et) with metamizole (met) on cell viability of canine D-17 and human U-2 OS osteosarcoma cell lines. * values below control in the Dunnett test.

Figure 2 shows the proliferative activity of the tested neoplastic cells exposed to doxorubicin at 1, 0.1, and 0.01 μg/mL, and cisplatin, carboplatin, and etoposide at 1 μg/mL combined with metamizole at 50, 5, and 0.5 μg/mL. 

It is noteworthy that metamizole at 50 μg/mL shows a cytoprotective or even stimulatory effect. However, further studies are needed to confirm its possible stimulatory activity in human and canine OSA cells. 

### 3.2. Apoptosis Analysis

**Figure 3:**
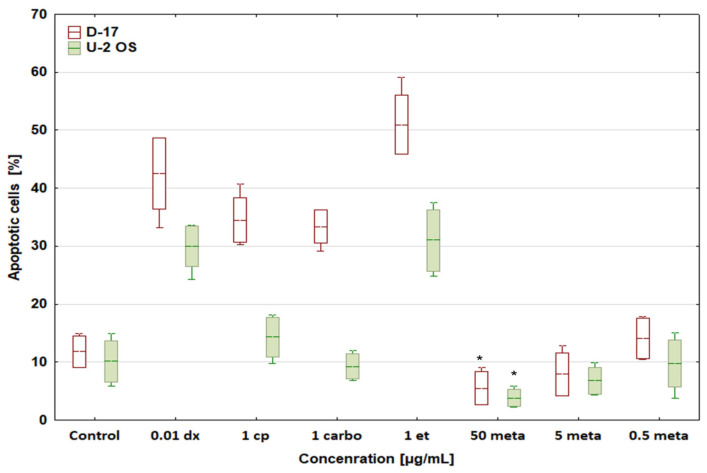
Effect of cytostatic drugs and metamizole on apoptosis in canine and human osteosarcoma cell lines. * values below control in the Dunnett test.

Our study showed that the strongest apoptosis-inducing effect in the canine OSA cell line (D-17) was observed after incubation of the cells with etoposide at 1 μg/mL and doxorubicin at 0.01 μg/mL (Figure 3). 

The percentage of apoptotic cells in the presence of these drugs was 50.96 ± 5.06% and 42.56 ± 6.11%, respectively. Doxorubicin (0.01 μg/mL) and etoposide (1 μg/mL) induced apoptosis of human osteosarcoma cells to a lesser extent (approx. 30%).

This study revealed a significant difference between the apoptosis induction rates triggered by platinum derivatives (cisplatin and carboplatin) in canine and human OSA lines. Cisplatin and carboplatin induced apoptosis in approximately 35% of D-17 cells and only in approximately 10% of U-2 OS cells. The percentage of apoptotic cells in both lines treated with metamizole at 50 μg/mL was below the control, which may suggest an anti-apoptotic effect of metamizole at low concentrations. Metamizole at a concentration below 5 μg/mL exerted no or only very weak effects (Figure 3)

**Figure 4:**
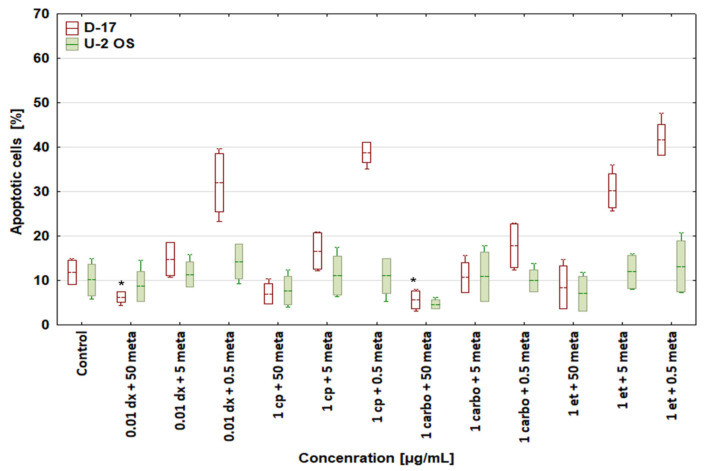
Effect of combinations of cytostatic drugs with metamizole on apoptosis in canine D-17 and human U-2 OS osteosarcoma cell lines. * values below control in the Dunnett test.

Metamizole at the highest tested concentration (50 μg/mL) attenuated the apoptosis of canine and human OSA cells induced by cytostatic drugs (doxorubicin, cisplatin, carboplatin, and etoposide) (Figure 4), but the strongest, most significant cytoprotective effects occurred in the D-17 cell line exposed to doxorubicin at 0.01 μg/mL and carboplatin at 1 μg/mL. 

### 3.3. Cell Cycle Analysis

**Figure 5:**
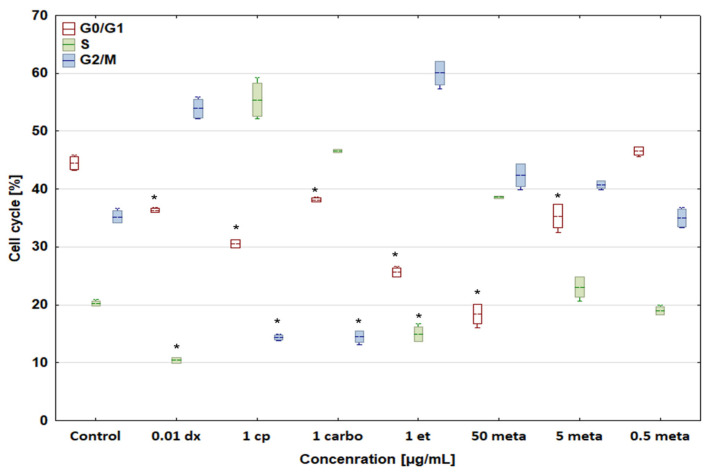
Effect of cytostatic drugs and metamizole on cell cycle in canine osteosarcoma D-17 cell line. * values below control in the Dunnett test.

**Figure 6:**
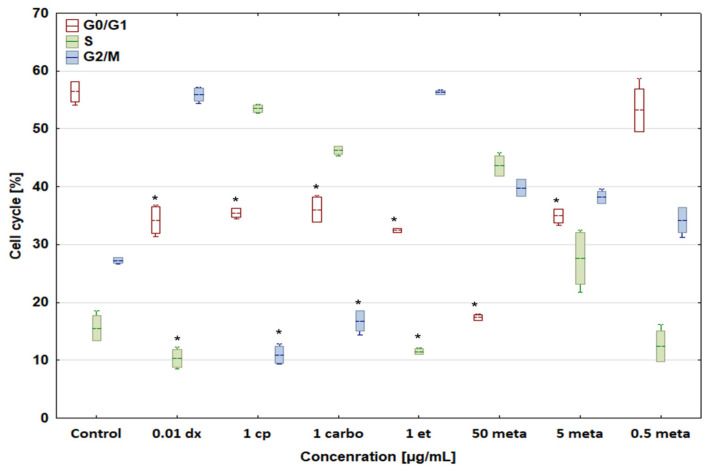
Effect of cytostatic drugs and metamizole on cell cycle in human osteosarcoma U-2 OS cell line. * values below control in the Dunnett test.

In comparison with the control, all concentrations of the tested drugs had the same effect on the cell cycle of both dog and human osteosarcoma cells (Figure 5 and Figure 6). Doxorubicin at a concentration of 0.01 μg/mL increased the percentage of cells in the G2/M phase to 53.93 ± 1.61 and 55.93 ± 1.15%, respectively, and lowered the number of cells in other phases. Cisplatin (1 μg/mL) and carboplatin (1 μg/mL), as cytostatic drugs with the same mechanism of action, had an identical effect on the cell cycle of both D-17 and U-2 OS cells. Both compounds enhanced the percentage of cells in the S phase to 55.42 ± 2.90 and 46.58 ± 0.27%, respectively, for canine osteosarcoma cells, and to 53.51 ± 0.66 and 46.27 ± 0.71%, respectively, for human osteosarcoma cells, while reducing the percentage of cells in the remaining G0/G1 and G2/M phases. Etoposide at 1 μg/mL increased the percentage of cells in the G2/M phase up to 60.11 ± 2.02% for canine osteosarcoma cells and 56.28 ± 0.37% for human osteosarcoma cells.

Metamizole at 50 μg/mL reduced the number of cells in the G0/G1 phase to 18.44 ± 1.63% (line D-17) and 17.40 ± 0.49% (line U-2 OS) and caused dose-dependent changes in the percentage of cells in the remaining phases of the cell cycle, with a visible tendency to return to values similar to those in the control samples.

**Figure 7:**
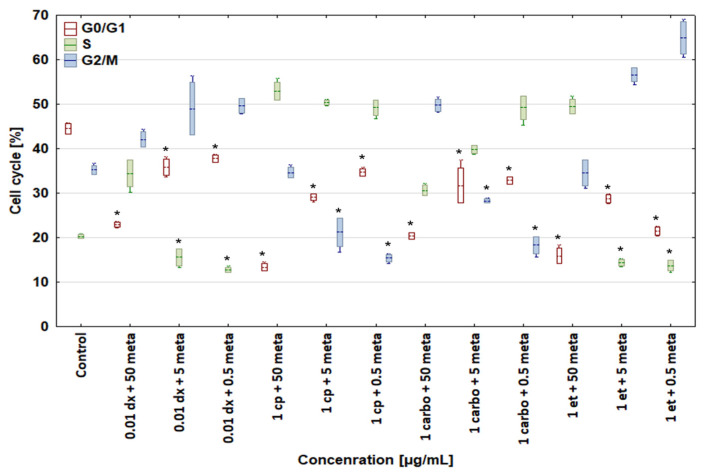
Effect of combinations of cytostatic drugs with metamizole on cell cycle in canine osteosarcoma D-17 cell line. * values below control in the Dunnett test.

**Figure 8:**
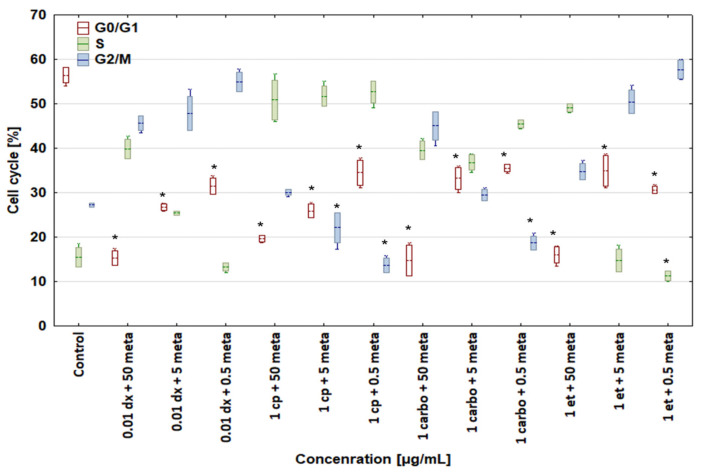
Effect of combinations of cytostatic drugs with metamizole on cell cycle in human osteosarcoma U-2 OS cell line. * values below control in the Dunnett test.

A combination of 0.01 μg/mL doxorubicin + 50 μg/mL metamizole increased the percentage of canine and human osteosarcoma cells in the S and G2/M phases up to 34.44 ± 3.06 and 39.78 ± 2.17% for the D-17 line and to 42.10 ± 1.73 and 45.64 ± 1.64% for the U-2 OS line, as compared with the control group, with a simultaneous reduction in cells in the G0/G1 phase (Figure 7 and Figure 8). In the samples treated with doxorubicin alone, a drop in the number of cells was seen in the S phase. The combinations of 1 μg/mL cisplatin + 50 μg/mL, 5 μg/mL, or 0.5 μg/mL metamizole enhanced the percentage of osteosarcoma cells in the S phase in both cell lines versus the control cells. The combination of 1 μg/mL carboplatin + 50 μg/mL metamizole increased the number of cells in the S phase of both the D-17 and U-2 OS lines up to 30.65 ± 1.13 and 39.56 ± 2.01%, respectively, as compared with the control. When the cell lines were treated with a combination of 1 μg/mL etoposide + 50 μg/mL metamizole, the percentage of cells in the S phase rose up to 49.47 ± 1.66 for the D-17 line and up to 49.11 ± 0.93% for the U-2 OS line above the control, and above the levels for etoposide or metamizole tested separately (Figure 5 and Figure 6). For all tested drugs and their concentrations, a significant decrease in the number of cells in the G0/G phase was seen in both cell lines.

## 4. Discussion

The viability analysis of D-17 and U-2 OS cells treated with metamizole revealed possible cytoprotective and anti-apoptotic properties of the drug. However, scientific reports on this matter are scarce. Our research is a pilot, informative, and very simple study investigating only two cell lines of one type of neoplasm. To confirm its results and hypothesis, wider studies should be carried out with normal cell lines, in silico experiments, or cell lines of other neoplasms of mesenchymal and epithelial origin. It is also noteworthy that the potential antagonising effect of metamizole on the cytotoxicity of doxorubicin, cisplatin, carboplatin, and etoposide is concentration-dependent.

The cytoprotective and anti-apoptotic effects of metamizole on HL-60, Jurkat, and Raji cells were observed in studies using UV radiation, arachidonic acid, and cycloheximide by Pompeia et al. [24]. Metamizole exhibits its anti-apoptotic effect at concentrations below 50 μg/mL, but above this value, it begins to show cytotoxic activity. This correlates with the results and conclusions drawn from our research, where metamizole at the highest tested concentration had a cytoprotective effect against both canine and human osteosarcoma cells, which resulted in reduced anticancer activity of all investigated cytostatic drugs. These findings obviously require further, more detailed research to determine metamizole’s mechanism of action. In our opinion, the cytotoxic effect of metamizole at 0.5 μg/mL on the D-17 line may have resulted from a procedural or methodological error and should not be taken into account in further assessments of the impact of metamizole on selected cell lines. Our study showed that metamizole at 50 μg/mL completely abolished the cytotoxic effect of doxorubicin on both canine and human osteosarcoma cells, but when the concentration of metamizole decreased, the cytotoxic effect of the tested drugs was “restored” to a greater or lesser extent.

Moreover, metamizole at 50 μg/mL attenuated the apoptosis induced by cytostatics in canine and human osteosarcoma cells. This effect was the strongest and most significantly cytoprotective in the D-17 cell line treated with 0.01 μg/mL doxorubicin and 1 μg/mL carboplatin. The remaining results are also worth considering, as metamizole applied together with doxorubicin, cisplatin, and carboplatin in the human osteosarcoma cell line seemed to confirm the validity of the hypothesis on its cytoprotective effect in cancer cells. The functional difference between the highest (50 μg/mL) and the lowest (0.5 μg/mL) tested concentration of metamizole was considerable and should be taken into account.

The cytoprotective effect of metamizole was concentration-dependent; it was most pronounced at a concentration below 100 μg/mL [25] and disappeared at lower concentrations. Dogs are more sensitive to the cytoprotective effects of metamizole than humans, as in dogs those effects already manifested at 40 μg/mL [26]. At metamizole concentrations of 5 and 0.5 μg/mL, the cytotoxic activity of the tested drugs returned to values close to the control. Due to its cytoprotective and anti-apoptotic effects [24], metamizole at high concentrations limited the ability of cytostatic drugs to induce apoptosis. Only when its concentration was lowered was the proapoptotic activity of the cytostatic drugs restored in canine osteosarcoma cells.

Moreover, using transcriptome profiling of a single patient’s tumour tissue with RNA-seq technology, Märtson et al. [27] identified adiponectin (ADIPOQ) as the most important and highly up-regulated gene in sarcoma. The specific genomics and genomic profiles that are essential for precision medicine need further analyses. Research confirms the viability of single-sample analysis, which is very useful in clinical conditions when fresh material is available [27]. Therefore, further studies can improve the translational potential of the described results and make them more applicable in medical and veterinary practice focused on single-patient samples. While OSA is still one of the most frequently diagnosed neoplasms in humans and animals and its aetiology is not fully known, novel biomarkers for OSA are urgently needed as they are essential for diagnosis and sufficient therapy. Exome sequencing in OSA cases and integrative analysis with whole-transcriptome RNA-seq data proved that the genes in which mutations were detected may be considered targets in the search for OSA biomarkers [28]. As single cases and DNA and RNA analyses are not enough to make decisive statements, future studies can confirm this hypothesis. The interdisciplinary and translational character of the above-mentioned findings shall be taken into account while designing new research projects.

Finally, we must mention studies combining clinical and molecular data on OSA [29], lung cancer [30], or even non-emphysematous chronic obstructive pulmonary disease [31]. These investigations have a high translational and practical value for further analyses. Our basic study on D-17 and U-2 OS cell line viability in the presence of metamizole and cytostatic drugs seems a valuable basis for both clinical and molecular research.

## 5. Conclusions

This study indicates that the combination of cytostatic and non-steroid anti-inflammatory drugs in OSA treatment should be used with caution. Metamizole at serum concentrations around 50 μg/mL shows strong antiapoptotic and cytoprotective effects in canine OSA (D-17 cell line). A similar activity of metamizole can also be observed in human OSA (U-2 OS cell line). Possible concentrations of metamizole in human serum are higher than in dogs; therefore, the drug can be more dangerous and cause more serious side effects during adjuvant chemotherapy.

## Figures and Tables

**Table 1:** Concentrations of drugs used in the experiment.

	Drug Name				
Concentration [μg/L]	Doxorubicin [dx]	Cisplatin [cp]	Carboplatin [carbo]	Etoposide [et]	Metamizole [meta]
	1	50	50	10	50
	0.5	20	20	5	20
	0.1	10	10	2.5	10
	0.05	5	5	1	5
	0.01	1	1	0.5	1
	0.005	0.5	0.5	0.1	0.5
					0.1
					0.05

**Table 2:** Combinations of cytostatic drugs with metamizole used in the experiment.

Drug Combinations [μg/mL]			
1 dx + 50 meta	1 cp + 50 meta	1 carbo + 50 meta	1 et + 50 meta
1 dx + 5 meta	1 cp + 5 meta	1 carbo + 5 meta	1 et + 5 meta
1 dx + 0.5 meta	1 cp + 0.5 meta	1 carbo + 0.5 meta	1 et + 0.5 meta
0.1 dx + 50 meta	0.1 cp + 50 meta	0.1 carbo + 50 meta	0.1 et + 50 meta
0.1 dx + 5 meta	0.1 cp + 5 meta	0.1 carbo + 5 meta	0.1 et + 5 meta
0.1 dx + 0.5 meta	0.1 cp + 0.5 meta	0.1 carbo + 0.5 meta	0.1 et + 0.5 meta
0.01 dx + 50 meta	0.01 cp + 50 meta	0.01 carbo + 50 meta	0.01 et + 50 meta
0.01 dx + 5 meta	0.01 cp + 5 meta	0.01 carbo + 5 meta	0.01 et + 5 meta
0.01 dx + 0.5 meta	0.01 cp + 0.5 meta	0.01 carbo + 0.5 meta	0.01 et + 0.5 meta
0.005 dx + 50 meta			
0.005 dx + 5 meta			
0.005 dx + 0.5 meta			
0.001 dx + 50 meta			
0.001 dx + 5 meta			
0.001 dx + 0.5 meta			

**Table 3:** EC_50_ values for the tested drugs [22].

EC_50_ Value [μg/mL]		
Drug Name	D-17	U-2 OS
Doxorubicin	0.056 ± 0.019 μg/mL	0.051 ± 0.003 μg/mL
Cisplatin	2.35 ± 0.43 μg/mL	2.38 ± 0.43 μg/mL
Carboplatin	6.45 ± 0.2 μg/mL	27.5 ± 2.3 μg/mL
Etoposide	6.27 ± 0.31 μg/mL	2.72 ± 0.51 μg/mL
Metamizole	>100 μg/mL	>100 μg/mL

## Data Availability

Data are contained within the article.
